# International Society of Neuropathology‐Haarlem Consensus Guidelines for Nervous System Tumor Classification and Grading

**DOI:** 10.1111/bpa.12171

**Published:** 2014-09-10

**Authors:** David N. Louis, Arie Perry, Peter Burger, David W. Ellison, Guido Reifenberger, Andreas von Deimling, Kenneth Aldape, Daniel Brat, V. Peter Collins, Charles Eberhart, Dominique Figarella‐Branger, Gregory N. Fuller, Felice Giangaspero, Caterina Giannini, Cynthia Hawkins, Paul Kleihues, Andrey Korshunov, Johan M. Kros, M. Beatriz Lopes, Ho‐Keung Ng, Hiroko Ohgaki, Werner Paulus, Torsten Pietsch, Marc Rosenblum, Elisabeth Rushing, Figen Soylemezoglu, Otmar Wiestler, Pieter Wesseling

**Affiliations:** ^1^ Department of Pathology Massachusetts General Hospital Harvard Medical School Boston MA USA; ^2^ Department of Pathology University of California San Francisco San Francisco CA USA; ^3^ Department of Pathology The Johns Hopkins University School of Medicine Baltimore MD USA; ^4^ Department of Pathology St. Jude Children's Research Hospital Memphis TN USA; ^5^ Department of Neuropathology Heinrich Heine University Duesseldorf Germany; ^6^ Clinical Cooperation Unit Neuropathology German Cancer Consortium (DKTK) German Cancer Research Center (DKFZ) Heidelberg Germany; ^7^ Department of Neuropathology Institute of Pathology Ruprecht‐Karls‐University Heidelberg Germany; ^8^ Department of Pathology Princess Margaret Hospital Toronto Canada; ^9^ Department of Pathology and Laboratory Medicine Emory University School of Medicine Atlanta GA USA; ^10^ Department of Pathology University of Cambridge Cambridge UK; ^11^ Department of Pathology and Neuropathology La Timone Hospital Aix Marseille University Marseille France; ^12^ Department of Pathology The University of Texas M. D. Anderson Cancer Center Houston TX USA; ^13^ Department of Radiological Oncological and Anatomo‐Pathological Sciences University La Sapienza Rome; ^14^ IRCCS Neuromed Pozzilli Italy; ^15^ Department of Laboratory Medicine and Pathology Mayo Clinic Rochester MN USA; ^16^ Department of Paediatric Laboratory Medicine The Hospital for Sick Children University of Toronto Toronto Canada; ^17^ Medical Faculty University of Zurich Switzerland; ^18^ Department of Neuropathology Heidelberg University Hospital Heidelberg Germany; ^19^ Department of Pathology Erasmus Medical Center Rotterdam The Netherlands; ^20^ Department of Pathology University of Virginia School of Medicine Charlottesville VA USA; ^21^ Department of Anatomical Pathology and Cellular Pathology The Chinese University of Hong Kong Hong Kong; ^22^ International Agency for Research on Cancer (IARC) Lyon France; ^23^ Institute of Neuropathology University Hospital Münster Münster Germany; ^24^ Institute of Neuropathology Brain Tumor Reference Center University of Bonn Bonn Germany; ^25^ Department of Pathology Memorial Sloan‐Kettering Cancer Center New York NY USA; ^26^ Institute for Neuropathology University Hospital of Zurich Zurich Switzerland; ^27^ Department of Pathology Hacettepe University Ankara Turkey; ^28^ German Cancer Research Center (DKFZ) Heidelberg Germany; ^29^ Department of Pathology VU University Medical Center Amsterdam The Netherlands; ^30^ Department of Pathology Radboud University Medical Center Nijmegen The Netherlands

**Keywords:** Astrocytoma, atypical teratoid rhabdoid tumor, brain tumors, classification, glioblastoma, glioma, grading, medulloblastoma, oligodendroglioma, World Health Organization

## Abstract

Major discoveries in the biology of nervous system tumors have raised the question of how non‐histological data such as molecular information can be incorporated into the next World Health Organization (WHO) classification of central nervous system tumors. To address this question, a meeting of neuropathologists with expertise in molecular diagnosis was held in Haarlem, the Netherlands, under the sponsorship of the International Society of Neuropathology (ISN). Prior to the meeting, participants solicited input from clinical colleagues in diverse neuro‐oncological specialties. The present “white paper” catalogs the recommendations of the meeting, at which a consensus was reached that incorporation of molecular information into the next WHO classification should follow a set of provided “ISN‐Haarlem” guidelines. Salient recommendations include that (i) diagnostic entities should be defined as narrowly as possible to optimize interobserver reproducibility, clinicopathological predictions and therapeutic planning; (ii) diagnoses should be “layered” with histologic classification, WHO grade and molecular information listed below an “integrated diagnosis”; (iii) determinations should be made for each tumor entity as to whether molecular information is required, suggested or not needed for its definition; (iv) some pediatric entities should be separated from their adult counterparts; (v) input for guiding decisions regarding tumor classification should be solicited from experts in complementary disciplines of neuro‐oncology; and (iv) entity‐specific molecular testing and reporting formats should be followed in diagnostic reports. It is hoped that these guidelines will facilitate the forthcoming update of the fourth edition of the WHO classification of central nervous system tumors.

## Introduction

The accurate classification of human neoplasms not only has implications for the care of individual patients (in estimating prognosis and guiding therapy) and for the conduct and interpretation of clinical studies of new diagnostic and therapeutic approaches, but also for the analysis and understanding of basic scientific experiments, for the elucidation of population‐based disease trends that may implicate environmental or other etiologies, and for the allocation of resources by governments and health insurers to support health care. Periodic revisions of tumor classifications therefore have diverse and important effects on many aspects of individual and population health. In recognition of this level of importance, over the past approximately half century, the World Health Organization (WHO) has sponsored tumor classifications by international experts. The last classification of tumors of the central nervous system emerged from a consensus meeting in 2006 1.

Over the past decade, insights into the molecular basis of human tumors have significantly improved both our biological understanding of neoplasms as well as our abilities to diagnose tumors and estimate their prognosis and likelihood of response to specific therapies. Brain tumors have shared in this molecular revolution, and in some areas have been at the forefront. Such molecular information can have diagnostic, prognostic and/or predictive value, with diagnostic and prognostic data often tightly linked with classification and grading, and predictive data also linked with the efficacy of particular therapeutic approaches. A critical question with major practical consequences has therefore arisen: how should clinically relevant molecular information be incorporated into nervous system tumor classification?

The 2007 WHO classification of tumors of the central nervous system included well over 100 distinct entities. Many of these tumors do not have molecular characteristics that at present require their implementation in the clinical diagnostic work‐up. However, other tumors are known to have clinically relevant molecular characteristics, and the basic question on how to incorporate this information optimally in their classification is similar across the entities. Before attempting to answer this question separately for each tumor entity, the major conceptual issues that underlie the question need to be addressed. Once the major conceptual issues are resolved, it would then be possible to use this information to guide the next WHO classification of individual tumor entities.

A proper answer to the above question also involves a balance between incorporating the latest molecular findings and the practical issues of clinical diagnosis and current patient management. One needs to identify the best balance between the most accurate, cutting‐edge molecular approaches and the everyday necessities of brain tumor patients and diagnostic laboratory resources, all of which are rapidly evolving. To do so requires the input of experts proficient in both the latest molecular advances and their clinical relevance as well as the most practical diagnostic procedures. In order to address the above question, we undertook a meeting of expert, molecularly oriented oncologic neuropathologists who had solicited input on this question from their neuro‐oncological colleagues (including neuro‐oncologists, medical oncologists, radiation oncologists, neurosurgeons, radiologists and other neuropathologists) prior to the meeting. The meeting addressed the critical question of how molecular information could be used for tumor classification as well as a series of related subquestions. The current publication outlines the consensus recommendations of that meeting.

## Methods

The meeting, entitled “WHO's Next?: A Colloquium to Guide Next Steps in Brain Tumor Classification and Grading,” was held from May 1 through May 3, 2014 in Haarlem, the Netherlands. Twenty‐eight neuropathologists from 10 different countries were invited, with only one unable to attend. In advance of the meeting, the participants were asked to canvass their colleagues involved in brain tumor diagnosis and therapy, both from their own institutions and from other institutions and clinical trial groups in which they participate. The group obtained input from over 150 neuro‐oncological specialists (greater than 50 adult and pediatric neuro‐oncologists, 40 neurosurgeons and 25 neuropathologists who did not participate in the meeting, as well as from about a dozen each of medical oncologists, neuroradiologists and radiation oncologists). In this manner, the participants provided their own views and those of colleagues from other disciplines at the meeting.

The meeting focused on the overall question and four subquestions shown in Table 1. The days were divided between plenary and breakout sessions, with the breakout sessions focusing on answering each subquestion in either adult or pediatric neoplasms. Within these large categories, the discussion was primarily focused on two groups of tumors in which the greatest progress has been made in unraveling molecular aberrations and that might serve as examples for how molecular information could be incorporated in general: (i) diffuse gliomas (including adult and pediatric astrocytoma, oligodendroglioma and glioblastoma); and (ii) embryonal tumors (including medulloblastoma and atypical teratoid/rhabdoid tumor).

**Table 1:** Questions

**Major question**
How can non‐histological criteria (eg, molecular, imaging, clinical, etc.) be used to enhance typing and grading of human brain tumors?
**Subquestions:**
1. What is the relationship between diagnosis and grade? Can tumor type and tumor grade be separated from one another, as occurs in other (non‐brain) tumor types? This also brings up the question of whether grade reflects natural history or likely prognosis after therapy.
2. How does one make recommendations about the use of molecular testing? Is molecular analysis required or optional? If optional, how does one formulate diagnoses to demonstrate this variability clearly? If required, does molecular diagnosis become incorporated into overall diagnosis or is it added as an extra level to the histological diagnosis? Does one make recommendations about the type of test to use? Does one make recommendations about specific cut‐off levels?
3. How does one formulate diagnoses if some institutions use molecular tests and others do not? If one uses molecular parameters to classify tumors, what does one call tumors that have the histological appearance but not the defining molecular feature? And what does one do with a tumor that has the defining molecular features of one tumor type, but the histologic appearance of another? In the era of broad sequencing/profiling, how does one classify a tumor with an unexpected but diagnostic mutation/profile?
4. Should one recommend the use of radiology and clinical parameters for typing and grading—keeping in mind that neuropathologists already occasionally use such features for classification (eg, location to diagnose medulloblastoma)?

The meeting began with an invited lecture by Dr. Daphne de Jong, who provided critical perspective on how hematopathologists have handled similar questions in their field. One of the key operational principles that the group unanimously agreed to adopt from the hematopathology experience is that clinically relevant disease entities should be defined as narrowly as possible in order to establish highly biologically uniform groups (eg, “B lymphoblastic leukemia/lymphoma with t(9:22)(q34;q11.2); *BCR‐ABL1*”), thereby excluding contaminating outliers that potentially confuse clinicopathological correlations. It was recognized that, with this approach, some tumors may not fit neatly into a single diagnostic category (eg, “aggressive B‐cell lymphoma with features intermediate between classic Hodgkin lymphoma and diffuse large B‐cell lymphoma”), but that this approach minimizes the risk of “wastebasket” diagnoses. In order to achieve this goal, entity definitions must thus be as precise and evidence‐based as possible.

The meeting was sponsored by the International Society of Neuropathology and made possible through generous support from the STOPbraintumors Foundation, the Netherlands. Neither of these organizations had a role in determining the recommendations made by the participants, but the executive committee of the International Society of Neuropathology reviewed and approved the plans for the meeting as well as the recommendations prior to publication.

## Meeting Conclusions

The meeting reached consensus regarding a broad set of conclusions that pertain to future classification systems for nervous system tumors, including the widely utilized WHO classification scheme. The group did not make comprehensive recommendations about individual tumor entities but, as shown below, utilized examples to illustrate how such decisions could be made in the future.

The conclusions reached can be summarized as follows:

(1)  Disease entities should be defined as precisely and objectively as possible in order to establish highly biologically and clinically uniform groups (ie, as previously undertaken by the hematopathology community). With this approach, some tumors may not fit into a diagnostic category and may require a descriptive diagnosis (eg, “diffuse glioma, not otherwise specified”); such “gray zone” tumors require further study before their exact position in the classification could be established.
(2)  Regarding the use of molecular information in diagnosis:
  (a)  Molecular information should be incorporated into the definitions of some diagnostic entities.
    (i)  For some entities, molecular information is required to provide an “integrated” diagnosis (see below) and only a descriptive histological diagnosis is acceptable if no molecular diagnostic testing is available. (See example of atypical teratoid/rhabdoid tumor, below. Note that this is an example only and that the eventual definitions will result from the WHO classification updating process.)
    (ii)  For other entities, molecular information will be necessary to provide an “integrated” diagnosis but a formal “NOS” (not otherwise specified) can be used if no molecular testing is performed. (See example of adult diffuse gliomas, below. Note that this is an example only and that the eventual definitions will result from the WHO classification updating process.)
    (iii)  To do the above, the definitions of some disease entities need to be refined, while others need to be added.
  (b)  For some diagnostic entities, histology alone will remain the basis for definition and diagnosis.
(3)  A key concept was that diagnoses should be “layered” in order to provide a format for displaying multiple types of information (Tables 2, 3, 4, 5). The analogy is to modern map technology, in which multiple layers can be readily superimposed on top of one another for easy viewing; such an approach has been advocated for disease taxonomy in general 2. A layered approach also facilitates standardization of diagnosis, which will be necessary to use such diagnostic information in computational systems. The suggested format is summarized in Table 2.
  (a)  The “integrated diagnosis” will be the top line in order to emphasize its primacy over the other lines even though it will be the last portion of the diagnostic format completed, as it is dependent on all diagnostic information being present. It is anticipated therefore that the integrated diagnosis will be “pending” for a period of time between histological examination and the availability of molecular information.
  (b)  The “histological classification” is the standard microscopic diagnosis that is based on hematoxylin and eosin staining and additional histological techniques such as histochemistry, immunohistochemistry and electron microscopy.
  (c)  The “WHO grade” is the standard histological grade. As in the past, WHO grade reflects natural history after surgery alone, rather than expected patient prognosis following current adjuvant therapies. For example, despite substantially improved control rates with current therapy, a medulloblastoma is still considered WHO grade IV as, if left without adequate postoperative treatment, it will follow a rapidly progressive, typically fatal course. Thus, the question arose as to whether a separate, additional grade reflecting expected behavior following therapy should be considered. However, because both current therapies and responses are subject to changes and having two different grades on a single report is confusing, the group opined that only a WHO grade based on natural history should be reported. This discussion raised the additional challenge that in some tumor types (eg, *IDH*‐mutant glioblastoma or WNT‐subgroup medulloblastoma in a child), stressing the WHO grade in the diagnosis may be more confusing than helpful, and that such situations may require a comment stating that the prognosis is better in such a molecular subtype than suggested by the grade.
  (d)  The “molecular information” is a synoptic account of the results of the molecular tests recommended for that particular tumor entity. Notably, the specific molecular tests recommended vary among tumor entities and will likely change over time. Moreover, the reporting of such molecular information should follow a set of guidelines, which are outlined in the following section.
(4)  Regarding molecular testing and reporting:
  (a)  Whether particular tests are required or recommended for diagnosis will depend on the biological properties of individual tumor types and whether the reported biomarkers are diagnostic, prognostic and/or predictive.
  (b)  Future decisions to incorporate such testing into diagnostic definitions will be based on conclusive published evidence from multiple independent studies.
  (c)  For some genetic tests, some general methodological approaches may be recommended over others (eg, detecting whole‐arm loss in oligodendrogliomas). In some situations, second‐level tests should follow first‐level tests (eg, *IDH1/2* sequencing to exclude rare mutations if IDH1 R132H immunohistochemistry is negative).
  (d)  In settings in which molecular testing is required or recommended, a report should state if it was not done (“unknown”) or if ordered (“pending”), along with a reason if not performed (eg, “tissue insufficient for molecular testing for *MGMT* promoter methylation status”).
  (e)  The methodological and results parameters of the assays performed should be included in reports in order to provide testing details and interpretive significance. This was felt important as, in some institutions, molecular reports are separate from surgical pathology reports and the results of molecular testing are either left out of the pathology report entirely or only abstracted in addenda. The group felt that by incorporating the pertinent details into the original surgical pathology report, this would facilitate comparability of data and multi‐institutional patient care, given that the pathology report alone is often forwarded to outside centers.
  (f)  Molecular testing must be based on histologically representative tissue. While this practice is routine in most academic centers, it remains possible that fragments of tissue are sent directly by neurosurgeons to molecular testing laboratories without histological confirmation; this practice risks false‐negative results and must be avoided.
(5)  The grading of adult type diffuse gliomas will follow standard, current WHO criteria for astrocytomas and oligodendrogliomas with the caveat that in some circumstances, assigning a precise grade is not possible. The latter is most relevant for the category of diffuse glioma that is not clearly of pure astrocytic or pure oligodendroglial subtype, either in the setting of a small biopsy in which selective sampling may be a concern or because of lack of molecular studies being performed, a discordance between morphology and molecular studies (eg, a histologically classic oligodendroglioma that lacks 1p/19q co‐deletion or shows ATRX loss), or a molecular pattern that does not fit neatly into a single tumor type. In such circumstances, the WHO grade may either be left off altogether (preferably with an explanatory comment) or may appear as “high grade” or “at least WHO grade …”. For example, a phenotypically ambiguous diffuse glioma with atypia, mitoses, microvascular proliferation, and necrosis could initially be diagnosed as being “at least WHO grade III” given that it would qualify as grade III if oligodendroglial (ie, anaplastic oligodendroglioma) or grade IV if astrocytic (ie, glioblastoma). In compliance with WHO terminology, the term “anaplastic” will precede any astrocytic or oligodendroglial tumor qualifying for a grade III designation. The term glioblastoma will be utilized for astrocytic neoplasms qualifying as grade IV.
(6)  Some pediatric tumor types will require separation from their adult histological “look‐alikes.” Separating these pediatric entities becomes critical now that there is clear evidence that the underlying molecular basis is different (eg, histone *H3.3 K27M* mutations in diffuse pediatric high‐grade gliomas/intrinsic pontine gliomas and a rarity of 1p/19q co‐deletion in pediatric oligodendrogliomas).
(7)  The inclusion of non‐tissue‐based information (eg, clinical, radiological information) is not required in the layered final diagnosis, but can be of clear utility in reaching a final diagnosis or determining sampling adequacy in individual cases. As such, non‐tissue‐based data can be included in the clinical history or comments section, as is already common practice.
(8)  Input from a broad constituency of clinical and scientific colleagues involved in neuro‐oncology is important in guiding future decisions regarding brain tumor classification.

**Table 2:** Report format

Layer 1: Integrated diagnosis (incorporating all tissue‐based information)
Layer 2: Histological classification
Layer 3: WHO grade (reflecting natural history)
Layer 4: Molecular information

**Table 3:** Diagnosis example: atypical teratoid/rhabdoid tumor

	A	B
Integrated diagnosis	Atypical teratoid/rhabdoid tumor, WHO grade IV	Embryonal tumor with rhabdoid features, WHO grade IV
Histological classification	Embryonal tumor with rhabdoid features	Embryonal tumor with rhabdoid features
WHO grade	IV	IV
Molecular information	INI1 loss of protein expression/mutation or BRG1 loss of protein expression/mutation	INI1 and BRG1 protein expression retained/not mutated or molecular/immunohistochemical testing not performed

In this example, using the layered diagnosis format, the integrated diagnosis of atypical teratoid/rhabdoid tumor is only possible in the setting of either INI1 or BRG1 loss of protein expression or mutation (column A); without these findings, only a descriptive diagnosis is possible (column B). (Note that this is an example only and that the eventual definition will result from the WHO classification updating process.)

**Table 4:** Reporting format example: medulloblastoma

Integrated diagnosis	Medulloblastoma histological subtype and molecular subgroup (eg, Wnt, SHH, non‐WNT/non‐SHH*), WHO grade IV
Histological classification	Classic, anaplastic, large cell, desmoplastic/nodular, medulloblastoma with extensive nodularity
WHO grade	IV
Molecular information	*MYC* amp, *NMYC* amp, *TP53* status, *CTNNB1* status, *SMO* status, *PTCH* status, i17q, monosomy 6**

Medulloblastoma diagnosis would incorporate the histological subtype, the WHO grade and the molecular subgroup.

*This is just an example of an approach to biological subgrouping and decisions on recommended subgrouping would await further deliberation by the WHO working group.

**This list of potentially detectable molecular alterations (presence vs. absence) is illustrative only and decisions on recommended tests would await further deliberation by the WHO working group.

Abbreviations: Amp = gene amplification; i17q = isochromosome or isodicentric chromosome 17q.

**Table 5:**
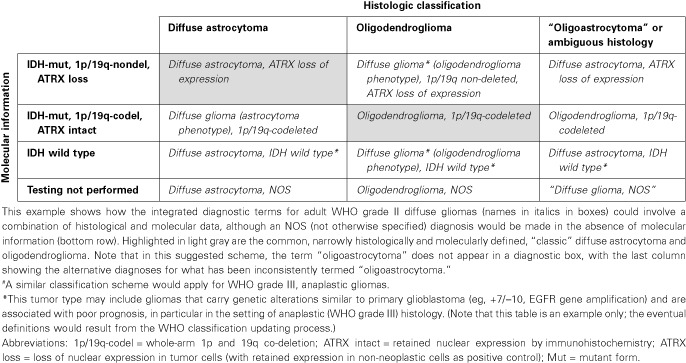
Example: integrated diagnoses for WHO grade II adult diffuse gliomas.^#^

## Meeting Recommendations/Guidelines

**Figure 1:**
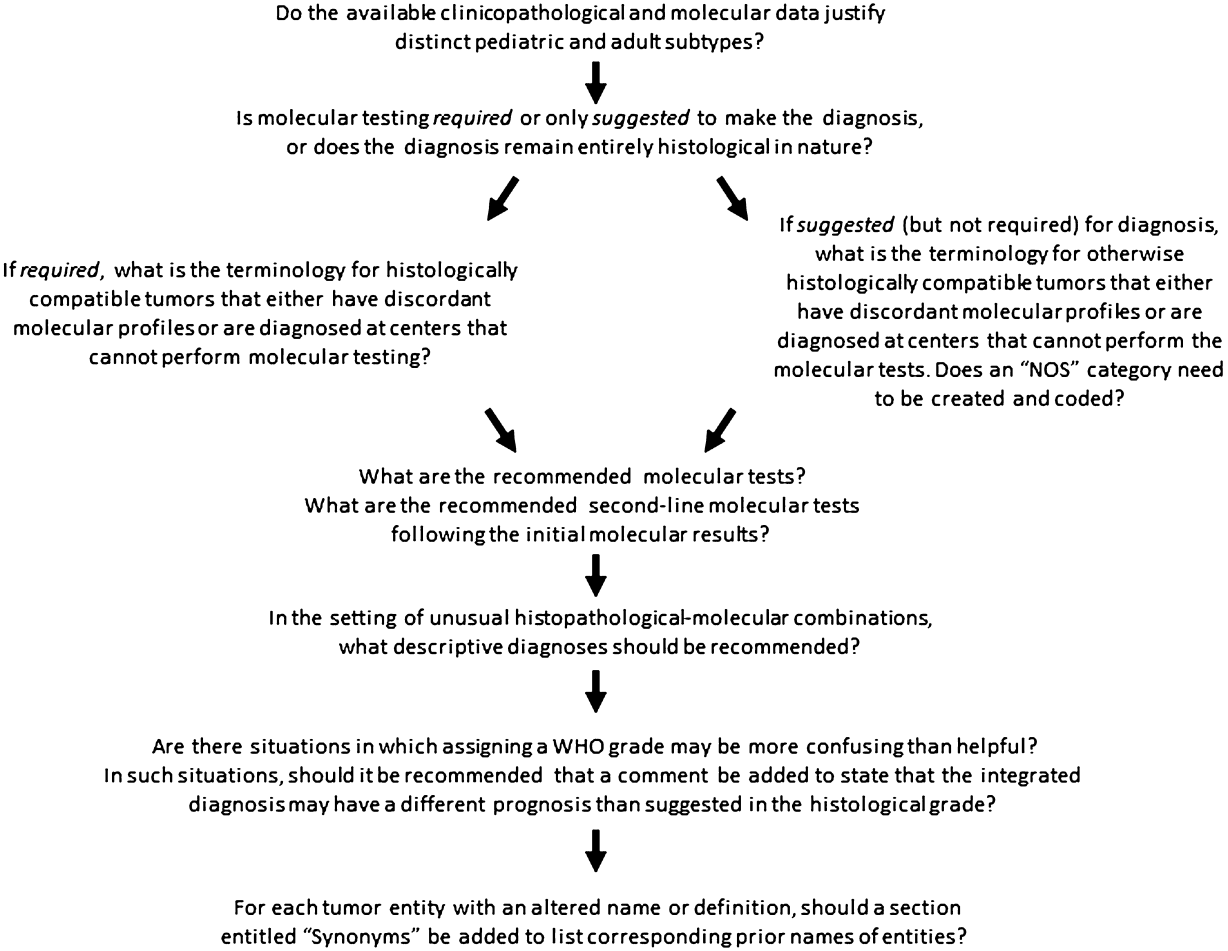
Suggested flow chart for classification decisions. A series of questions can guide each entity‐related working group through key decisions that will determine the role of molecular information in formulating diagnostic entities.

The meeting did not address the above issues across the spectrum of tumor entities in the 2007 WHO classification, but instead addressed these issues in “test” cases, which included the diffuse gliomas, medulloblastoma and atypical teratoid/rhabdoid tumor (AT/RT). Nonetheless, the group felt that the principles derived from analyzing these tumor types could be applied across the spectrum of WHO entities in the forthcoming update of the fourth edition. As such, the group proposes the following guidelines with a decision flowchart included as Figure 1.

(1)  For each tumor that involves a wide age spectrum, WHO working groups should decide whether the available molecular and clinicopathological data justify separating it into distinct pediatric and adult subtypes. Decisions on suggested clinical‐radiological phenotypes and defining molecular features would be necessary. In some situations, temporary “gray zone” designations may be needed.
(2)  For each tumor entity, the working groups should decide whether molecular testing is required or suggested to make the diagnosis, or whether clinically meaningful diagnostic or prognostic molecular information is lacking, such that the diagnosis remains entirely histological in nature.
  (a)  For those entities for which molecular information is required for diagnosis, terminology should be considered for otherwise histologically compatible tumors that have discordant molecular profiles, lack sufficient tissue for molecular analyses or are diagnosed at centers that cannot perform molecular testing. In most of the latter situations, it is expected that only a descriptive diagnosis will be possible and that such descriptive diagnoses may not have distinct International Classification of Diseases for Oncology (ICD‐O) codes (eg, embryonal tumor with rhabdoid features or “mixed/ambiguous” diffuse glioma of indeterminate phenotype).
  (b)  For those entities for which molecular diagnostic information is suggested (but not required) for diagnosis, terminology should be considered for otherwise histologically compatible tumors that either have discordant molecular profiles or are diagnosed at centers that cannot perform the molecular tests. In such situations, it is likely that “NOS” (not otherwise specified) categories will need to be created and that such “NOS” diagnoses will need to have distinct ICD‐O codes (eg, oligodendroglioma, NOS for which testing is not performed).
(3)  For each tumor entity, the working groups should make recommendations for situations in which clarification of the WHO grade may be helpful. For example, as WHO grade reflects natural history following surgery alone, a “grade IV” designation in an *IDH*‐mutant glioblastoma may be somewhat misleading in terms of expected behavior. In such situations, it can be helpful to add a comment stating that the prognosis may be better than suggested by the grade.
(4)  For each tumor entity with an altered name or definition, the working groups should consider the addition of a section entitled “synonyms” in order to list the corresponding prior names of entities, thus allowing for ready connection between the updated and prior classification systems for clinical care as well as clinical, experimental and epidemiological study purposes. For instance, if the diagnosis of “oligoastrocytoma” is no longer rendered, the synonym “oligoastrocytoma” would be listed for histologically ambiguous diffuse gliomas, whether or not molecular testing has resolved tumor diagnosis.
(5)  For each group of tumors (eg, the diffuse gliomas), considerations should be given to defining recommended tests and the order of carrying out such tests if done sequentially. For example, in a diffuse glioma without morphological evidence of oligodendroglioma, *IDH1* and *ATRX* analysis may be carried out initially, but would 1p/19q analysis still need to be performed in gliomas with *ATRX* mutation or loss of protein expression (ie, molecular evidence of astrocytoma)? When a diffuse glioma has whole‐arm 1p/19q co‐deletion (ie, molecular evidence of oligodendroglioma), is it still necessary to assess the *IDH* and *ATRX* status as well? Another major question would be whether sequencing analysis for *IDH* mutation is needed when IDH1 R132H immunohistochemistry is negative. One might argue in such a situation that additional sequence analysis would be recommended in the setting of a low‐grade tumor in a young adult patient in which the likelihood of *IDH* mutation is high, but not in the setting of an elderly patient with a glioblastoma that had no prior history or histological evidence of a lower grade precursor (ie, a clinically and histologically classic primary glioblastoma). It is also entirely possible that genetic tests not discussed at this meeting (eg, *TERT* mutation) will be incorporated into diagnostic definitions at the time of the eventual WHO classification revisions. Lastly, it may be that certain important “negative” findings should be included, for example, that amplification of the chromosome 19 miRNA cluster was not found in the evaluation of an embryonal tumor.
(6)  It is expected that a combined histological and molecular approach can classify the vast majority of cases and all of the common combinatorial scenarios. Nonetheless, there will likely be unusual combinations that will necessitate descriptive diagnoses and the working groups should address recommendations on how to list these rare situations.
(7)  The WHO working groups should avail themselves of the broad input that will be solicited from the clinical and scientific neuro‐oncology communities.

## Summary

As data has become more plentiful and complex in the modern world, there has been a need to distinguish among data, information, knowledge and wisdom (“DIKW”) and to develop systems that facilitate the conversion of data to information to knowledge and eventually to wisdom. Similarly, in medicine, as data have become more plentiful and more complex, there is an increasing challenge to convert that data into clinically meaningful information that can be used to treat patients. Presenting that data in logical and accepted formats is a *sine qua non* in order to use that data and information to generate knowledge. The ISN‐Haarlem guidelines provide logical formats to convey tissue‐based data and information. As such, it is hoped that these templates be used by the various WHO working groups as they begin defining and redefining nervous system tumor entities and that these definitions form the basis for the knowledge that will improve the treatment of brain tumor patients in the near future.
